# Ag nanoparticles/PPV composite nanofibers with high and sensitive opto-electronic response

**DOI:** 10.1186/1556-276X-6-121

**Published:** 2011-02-07

**Authors:** Jinfeng Chen, Peipei Yang, Chunjiao Wang, Sumei Zhan, Lianji Zhang, Zonghao Huang, Wenwen Li, Cheng Wang, Zijiang Jiang, Chen Shao

**Affiliations:** 1 Faculty of Chemistry, Northeast Normal University, Changchun, 130024, People's Republic of China; 2 Bilingual Teaching Training Center, Changchun Normal University, Changchun 130032, People's Republic of China; 3 Faculty of Chemistry and Materials Science, Heilongjiang University, Harbin 150080, People's Republic of China

## Abstract

The novel Ag nanoparticles/poly(*p*-phenylene vinylene) [PPV] composite nanofibers were prepared by electrospinning. The transmission electron microscope image shows that the average diameter of composite fibers is about 500 nm and Ag nanoparticles are uniformly dispersed in the PPV matrix with an average diameter of about 25 nm. The Fourier transform infrared spectra suggest that there could be a coordination effect to a certain extent between the Ag atom and the π system of PPV, which is significantly favorable for the dissociation of photoexcitons and the charge transfer at the interface between the Ag nanoparticle and the PPV. The Au top electrode device of the single Ag/PPV composite nanofiber exhibits high and sensitive opto-electronic responses. Under light illumination of 5.76 mW/cm^2 ^and voltage of 20 V, the photocurrent is over three times larger than the dark current under same voltage, which indicates that this kind of composite fiber is an excellent opto-electronic nanomaterial.

## Introduction

Recently, 1D opto-electronic nanomaterials, especially the 1D organic opto-electronic nanomaterials, have received much attention of scientists because of their distinctive geometries, novel opto-electronic properties, and the potential application in nano/micro devices [1-5].

Electrospinning is an efficient technique for the fabrication of 1D polymer-based nanomaterials. Up to now, a lot of polymers and polymer-based composite materials have been fabricated by electrospinning [5-7]. Poly(*p*-phenylene vinylene) [PPV] is a typical conjugated polymer which has good photoluminescent [PL] and electroluminescent properties as well as photovoltaic and nonlinear optical properties [8-10]. Our research group has successfully fabricated the PPV nanofibers and the PPV-based composite nanofibers by electrospinning, such as TiO_2_/PPV and CdSe/PPV nanofibers, etc., which showed novel opto-electronic properties [11,12].

Metal nanomaterials exhibit many novel physical and chemical characteristics which arise from their quantum confinement effects and their enormously large specific surface areas. Therefore, metal nanomaterials are used as a kind of block to build advanced functional materials or to improve the efficiency of devices in many researches. Lee et al. [13] reported that the incorporation of gold nanodots on the indium tin oxide surface can obviously increase the power conversion efficiency of poly(3-hexylthiophene)/[6][6]-phenyl C61-butyric acid methyl ester solar cell. Nah et al. [14] reported that the electrochromic absorption was markedly enhanced in Ag nanoparticles embedded in MEH-PPV composite films. The opto-electronic response of the pristine PPV film device is relative low [10], which makes the investigation of the opto-electronic character of a single PPV nanofiber difficult. We expect that incorporating Ag nano-particles in PPV nanofibers can prepare a novel composite nanofiber with a high opto-electronic response.

In this paper, Ag nanoparticles/PPV composite nanofibers were successfully prepared by electrospinning. Then, the Au top electrode device of a single composite nanofiber was fabricated on a SiO_2 _substrate by an 'organic ribbon mask' technique, which showed high and sensitive opto-electronic response.

## Experimental

### Preparation of Ag nanoparticles and Ag/PPV composite nanofibers

Sodium borohydride (NaBH_4_) was purchased from Sinopharm Chemical Reagent Co., Ltd. (Shanghai, China), while ethanol and silver sulfate (Ag_2_SO_4_) were from Beijing Beihua Fine Chemicals Co., Ltd. (Beijing, China). All reagents were of analytical grade and used without further purification.

**Figure 1:**
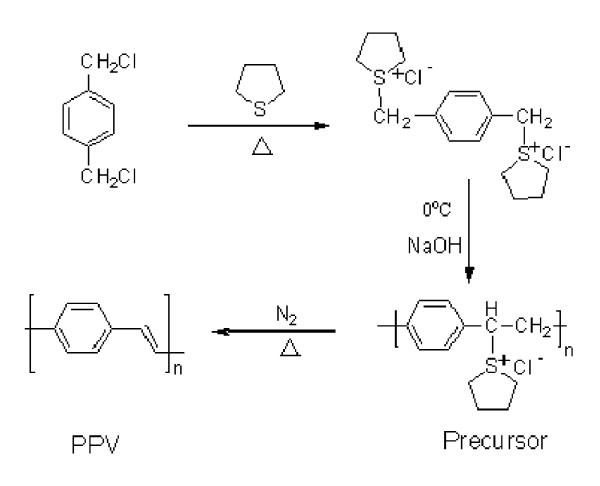
**The synthesis route of PPV**.

The synthesis route of PPV is presented in Figure 1, and the PPV precursor ethanol solution (0.4 wt.%) was prepared according to [15]. Ag nanoparticles were prepared by the reduction of silver ions in Ag_2_SO_4 _with NaBH_4_.

The synthesis process of Ag nanoparticles/PPV composite nanofibers is as follows: Firstly Ag_2_SO_4 _(0.21 g, 0.67 mmol) was dissolved in 100 ml distilled water to get a clear solution, and then NaBH_4 _(1.40 g, 0.037 mol) was added into the solution with vigorous stirring under N_2_-saturated atmosphere. After filtering, drying, and triturating, we obtained Ag nanoparticles. Then, 3 mg of Ag nanoparticles was added into 0.4 wt.% PPV precursor ethanol solution (2.63 g) with stirring at room temperature for 24 h to obtain a new solution. Then, the solution was electrospun at room temperature, with positive voltage of 15 kV, humidity of 45%, and tip-to-collector distance of 20 cm. Finally, the electrospun fibers were heated at 180°C for 4 h in a vacuum oven for conversion of the PPV precursor to PPV.

The pristine PPV nanofibers were also prepared in a similar procedure as described above.

### Characterization

The small-angle X-ray diffraction [SAXRD] measurements were performed on a small-angle X-ray diffractometer (PX13-010, Japan). Fourier transform infrared [FTIR] measurements were carried out on a Fourier transform infrared spectrometer (Magana 560, Nicolet Corp., Madison, WI, USA). The photoluminescence excitation [PLE] and PL measurements were made on an Eclipse Fluorescence Spectrophotometer (Varian Corp., Palo Alto, CA, USA). The morphology of nanofibers was observed using a transmission electron microscope [TEM] (FP 5021/20, Czech Republic). A scanning electron microscope [SEM] (ESEM XL-30, FEI Company, Hillsboro, OR, USA) was used to reveal the structure of the Au top electrode device of a single Ag/PPV composite nanofiber. The opto-electronic response of this device was measured with a Keithley 4200 SCS and a Micromanipulator 6150 probe station in a clean and shielded box.

## Results and discussion

### SAXRD patterns

**Figure 2:**
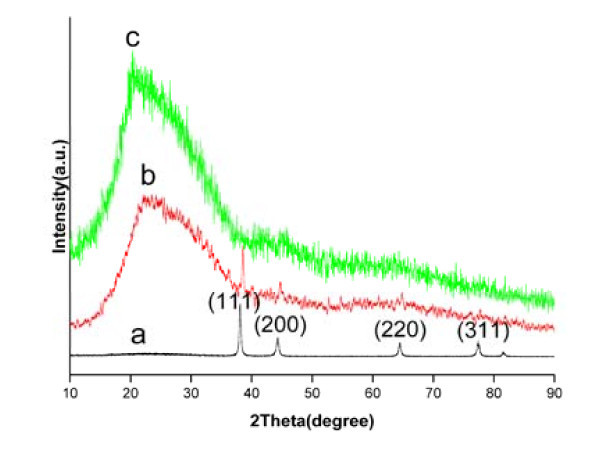
**XRD patterns**. *a *Ag nanoparticles, *b *Ag/PPV composite fibers, *c *pure PPV fibers.

The SAXRD patterns of Ag nanoparticles, Ag/PPV composite nanofibers, and pure PPV nanofibers are shown in Figure 2, using CuKα radiation (*λ *= 0.154060 nm). The same diffraction peaks at 38.13°, 44.23°, 64.48°, and 77.33°in the SAXRD patterns of Ag particles and Ag/PPV composite fibers can verify the generation of highly crystalline Ag nanoparticles with the face-centered cubic crystal structure (JCPDS card no. 04-0783).

### TEM image

**Figure 3:**
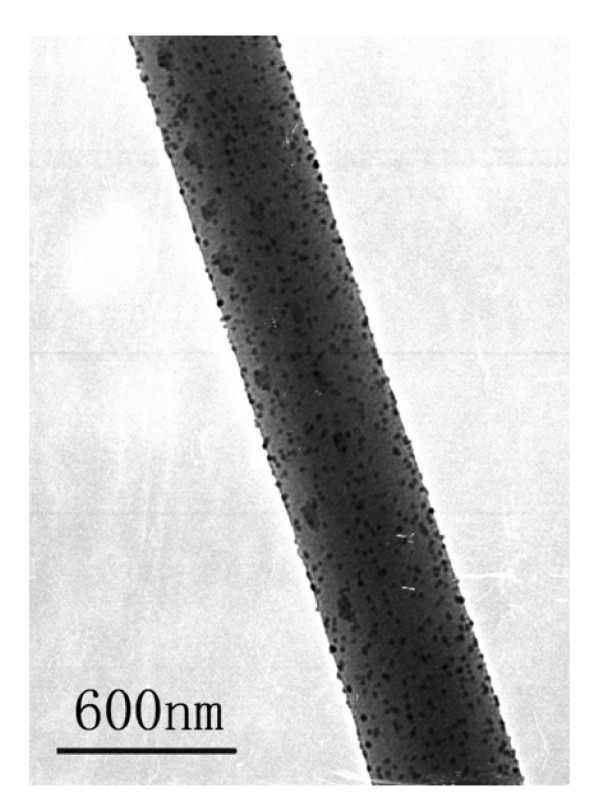
**TEM image of an Ag/PPV composite nanofiber**.

The TEM image of an Ag/PPV composite fiber (Figure 3) shows that the fiber diameter was about 500 nm and the average diameter of Ag nanoparticles was about 25 nm. During electrospinning, the Coulomb repulsion among charged Ag nanoparticles should be the main factor making the Ag nanoparticles uniformly dispersed.

### FTIR spectra

**Figure 4:**
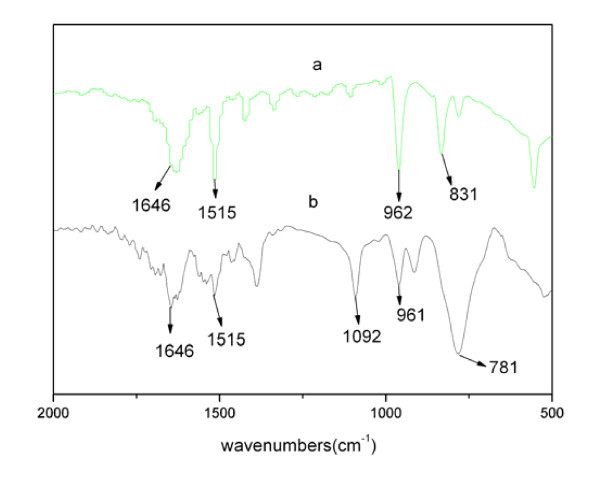
**FT-IR spectra**. *a *Pure PPV fibers, *b *composite fibers.

From the FTIR spectra of pristine PPV fibers and composite fibers (Figure 4), we can conclude that both pristine PPV fibers and composite fibers have the three similar characteristic absorption peaks at 1,646 cm^-1 ^(C = C bond stretching mode), 1,515 cm^-1 ^(C-C ring stretching mode), and 962 cm^-1 ^(*trans*-vinylene C-H out-of-plane bending mode), which implies that the conjugation structure of PPV is basically kept in composite fibers. However, the characteristic absorption peak of the pristine PPV fibers at 831 cm^-1 ^(*p*-phenylene C-H out-of-plane bending mode) was obviously broadened in the spectrum of composite fibers. This phenomenon could be explained by the coordination effect to a certain extent between the 5*S *orbital of the Ag atom, locating over the conjugation plane of PPV, and the π system of PPV (especially the π system of the benzene ring part). Therefore, the SP^3 ^hybrid orbital component could be partly introduced into the C-H bond (SP^2 ^hybrid orbital) of the benzene ring. The occurrence of the coordination effect should be significantly favorable for the charge separation of photoexcitons and the charge transfer at the interface between Ag nanoparticles and PPV so as to obviously improve the opto-electronic response of the composite materials.

### PL spectra

**Figure 5:**
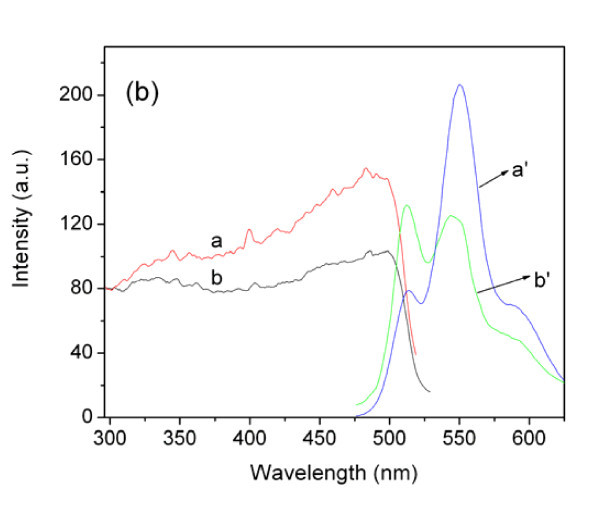
**PL (*λ*_ex _= 350 nm) and PLE (*λ*_em _= 550 nm) spectra**. *a *Pure PPV nanofibers, *b *composite nanofibers.

Figure 5 shows the PL (*λ*_ex _= 350 nm) and PLE (*λ*_em _= 550 nm) spectra of the pristine PPV nanofibers and the composite nanofibers. The positions of the PPV characteristic peaks (at 515 and 550 nm) did not change, which indicates that the functional structure of PPV in the composite fibers is kept, which is consistent with the FTIR result. However, in the composite fibers' spectrum, the relative enhancement of 515-nm emission peaks, compared with the 550-nm emission peaks, indicates that the addition of Ag nanopartilces decreases the reabsorption among PPV chains.

### Opto-electronic characteristics of the single composite fiber device

**Figure 6:**
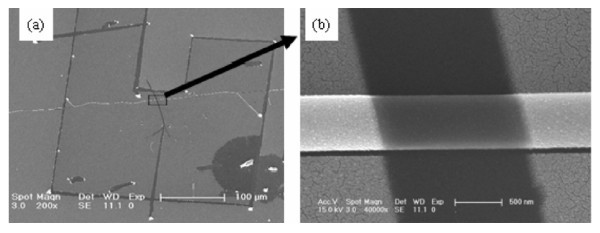
**top electrode device of the single composite fiber**. **a **SEM image of Au top electrode device (the part closed by polygon indicated by the *red arrows*). **b **SEM image of the device's channel (insulate gap) and crossed nanofiber.

To measure the opto-electronic property of the composite nanofiber, the novel 'organic ribbon mask' technique of Professor Hu's group [16] was used to construct the Au top electrode device of the single composite fiber shown in Figure 6a,b. The fabrication process of the device is briefly described below: Firstly, a single composite nanofiber was transferred onto the SiO_2 _substrate (the white line and the horizontal fiber in Figure 6a,b, respectively). Then, an organic ribbon with a diameter of approximately 1.5 μm was picked up and crossed over the composite nanofiber. Finally, the gold was vacuum-deposited. After the 'organic ribbon mask' was peeled off, the insulate part acted as the channel of device (see the dark part in Figure 6b). Finally, the insulate lines were drawn on the Au film using a micromanipulator probe to form a polygon (indicated by the arrows in Figure 6a), which was the device outline.

**Figure 7:**
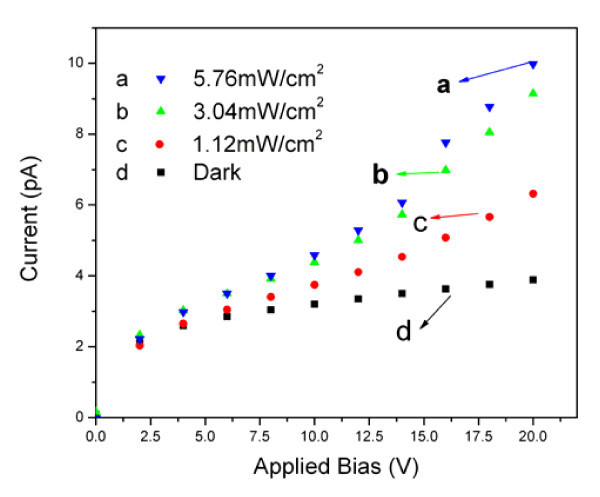
**Photocurrent of the composite nanofiber**. *J-V *curves of the single Ag/PPV composite nanofiber under light illumination with different intensities.

The *I-V *characteristics of the device were measured under light illumination from a Xe lamp with different intensities at room temperature in the shielded box. Figure 7 shows that the photocurrent of the composite nanofiber obviously increases with increasing the light intensity from 0 to 5.76 mW/cm^2^. The *I-V *curves in Figure 7 show the non-ohmic character, which is consistent with the *I-V *curves of PPV and its composite film devices [17]. Under light illumination of 5.76 mW/cm^2 ^and voltage of 20 V, the photocurrent is over three times larger than the dark current, which indicates that the composite nanofibers have high and sensitive opto-electronic response. The reason for the improvement (or enhancement) of the opto-electronic response of the composite nanofiber should be attributed to the following factor: there is the coordination effect to a certain extent between the Ag atom and the π system of PPV mentioned in "FTIR spectra."

It is noticed that the two *I-V *curves under light illumination of 3.04 and 5.76 mW/cm^2 ^are close to each other before 10 V, e.g., the phenomenon of photoresponse saturation happens, which could be related to the charge accumulation and de-trapping by light effects at the contacts.

## Conclusions

The Ag nanoparticles/PPV composite nanofibers with an average diameter of 500 nm were prepared by electrospinning. The TEM image shows that the Ag nanoparticles with an average diameter of 25 nm were dispersed uniformly in the PPV matrix. It was deduced from the FTIR spectra that there was a complexation between the Ag atom and the π system of PPV, which should be significantly favorable for the charge separation of photoexcitons and the charge transport at the interface between the Ag particles and the PPV. The *J-V *measurement of the device under light illumination with different intensities shows that the Ag nanoparticles/PPV composite nanofibers have high and sensitive opto-electronic response and will have good potential application in the micro/nano organic opto-electronic field.

## Competing interests

The authors declare that they have no competing interests.

## Authors' contributions

JC and PY carried out the preparation of the fibers and the devices. CW drafted the manuscript. SZ and LZ participated in sequence alignment. ZH contrubited to the redaction of the manuscript and in the design of the study. WL and CW and CS participated in the certain measurements. All authors read and approved the final manuscript.

## References

[B1] Gu FX Zhang L Yin XF Tong LM Polymer single-nanowire optical sensors Nano Lett 2008 8 2757 10.1021/nl8012314 18672942

[B2] Yu KH Chen JH Enhancing Solar Cell Efficiencies through 1-D Nanostructures Nanoscale Res Lett 2009 4 1 10.1007/s11671-008-9200-y20596408

[B3] Kjelstrup-Hansen J Norton JE da Silva Filho DA Brédas J-L Rubahn H-G Charge transport in oligo phenylene and phenylene-thiophene nanofibers Org Electron 2009 10 1228 10.1016/j.orgel.2009.06.015

[B4] Chang M-Y Wu C-S Chen Y-F Hsieh B-Z Huang W-Y Ho K-S Hsieh T-H Han Y-K Polymer solar cells incorporating one-dimensional polyaniline nanotubes Org Electron 2008 9 1136 10.1016/j.orgel.2008.08.001

[B5] Li D Xia YN Fabrication of titania nanofibers by electrospinning Nano Lett 2003 3 555 10.1021/nl034039o

[B6] Li ZY Huang HM Wang C Electrostatic Forces Induce Poly(vinyl alcohol)-Protected Copper Nanopartocles to Form Copper/Poly(vinyl alcohol) Nanocables via Electrospinning Macromol Rapid Commun 2006 27 152 10.1002/marc.200500627

[B7] Xin Y Huang ZH Peng L Wang DJ Photoelectric performance of poly(p-phenylene vinylene)/Fe_3_O_4 _nanofiber array J Appl Phys 2009 105 086106 10.1063/1.3116552

[B8] Burroughes JH Bradley DDC Brown AR Marks RN Mackay K Friend RH Burns PL Holmes AB Light-emitting diodes based on conjugated polymer Nature 1990 347 539 10.1038/347539a0

[B9] Prasad PN Williams DJ Introduction to Nonlinear Optical Effect in Molecules and Polymers 1991 New York: Wiley 284

[B10] Marks RN Halls JJM Bradley DDC Friend RH Holmes AB The photovoltaic response in poly(p-phenylene vinylene) thin-film device J Phys Condens Matter 1994 6 1379 10.1088/0953-8984/6/7/009

[B11] Jiang ZJ Huang ZH Yang PP Chen JF Xin Y Xu JW High PL-efficiency ZnO nanocrystallites/PPV composite nanofibers Compos Sci Technol 2008 68 3240 10.1016/j.compscitech.2008.08.010

[B12] Wang C Yan EY Huang ZH Zhao Q Xin Y Fabrication of Highly Photoluminescent TiO2/PPV Hybrid Nanoparticle-Polymer Fibers by Electrospinning Macromol Rapid Commun 2007 28 205 10.1002/marc.200600626

[B13] Lee JH Park JH Kim JS Lee DY Cho K High efficiency polymer solar cells with wet deposited plasmonic gold nanodots Org Electron 2009 10 416 10.1016/j.orgel.2009.01.004

[B14] Nah YC Kim SS Park JH Park HJ Jo J Kim DY Enhanced electrochromic absorption in Ag nanoparticle embedded conjugated polymer composite films Electrochem Commun 2007 9 1542 10.1016/j.elecom.2007.02.009

[B15] Halliday DA Burn PL Friend RH Bradley DDC Holmes AB Determination of the average molecular weigth of poly(*P*-phenylenevinylene) Synthetic Met 1993 55 902 10.1016/0379-6779(93)90172-S

[B16] Jiang L Gao JH Wang EJ Li HX Wang ZH Hu WP Jiang L Organic Single-Crystalline Ribbons of a Rigid "H"-type Anthracene Derivative and High-Performance, Short-Channel Field-Effect Transistors of Individual Micro/Nanometer-Sized Ribbons Fabricated by an "Organic Ribbon Mask" Technique Adv Mater 2008 20 2735 10.1002/adma.200800341 25213898

[B17] Álvaro M Corma A Ferrer B Galletero MS García H Peris E Increasing the Stability of Electroluminescent Phenylenevinylene Polymers by Encapsulation in Nanoporous Inorganic Materials Chem Mater 2004 16 2142

